# A Note on the Treatment of Wounds of the Genital Organs in Warfare

**Published:** 1919

**Authors:** 


**A Note on the Treatment of Wounds of the Genital Organs in Warfare.** By CHARLES GREENE CUMSTON. *Annals of Surgery*, September, 1918.

There may be merely a contusion of the scrotum, or a simple wound, with or without some foreign body lodged in its cavity, with or without lesions of the urethra or hernia of the testicle.

The seminal gland may be simply contused or partially or totally destroyed.

Wounds of the scrotum are recovered from with ease, and this also applies to traumatic orchitis. However, when hernia of the testicle arises, three eventualities are to be looked for: (1) the organ may slough; (2) become reduced spontaneously; (3) become grafted on a neighboring area.

When the testicle is injured to an extent beyond repair, castration must be done.

In contusion of the scrotum, moist humid dressings applied with very slight compression are usually quite sufficient to subdue the pain and tumefaction. If a hematoma of the scrotum or a hematocele of the vaginalis are suspected, an incision should be performed. In cases of large loss of scrotal tissue, skin grafts must be made.

If a missile or foreign body is lodged in the scrotal cavity it should be removed at once. When the testicle is untouched or only slightly contused, the only rational treatment is its reduction into the bursa and suture of the latter. The reduction may be delayed for a few days until the scrotal wound has been properly cleansed, but at the same time the vitality of the testicle must be carefully watched.

Admitting that the testicle and its vessels are intact, irreducibility is never an indication for primary castration. There is every reason to attempt reduction, even when the testicle is contused or offers a superficial wound.

Not infrequently, neuralgic paroxysms have their starting point in the testicular stump, so that an interference for its relief is indicated if the fellow organ is healthy.

Suprapubic cystotomy should be done for retention of urine following an injury to the urethra, and a few days later the urethra can be repaired by some of the many methods at our disposal.

